# Diagnosis of vascular cognitive impairment: recommendations of the scientific department of cognitive neurology and aging of the Brazilian Academy of Neurology

**DOI:** 10.1590/1980-5764-DN-2022-S104PT

**Published:** 2022-11-28

**Authors:** Breno José Alencar Pires Barbosa, José Ibiapina Siqueira, Gilberto Sousa Alves, Felipe Kenji Sudo, Claudia Kimie Suemoto, Fernanda Tovar-Moll, Jerusa Smid, Lucas Porcello Schilling, Marcio Luiz Figueredo Balthazar, Norberto Anízio Ferreira Frota, Leonardo Cruz de Souza, Francisco Assis Carvalho Vale, Paulo Caramelli, Paulo Henrique Ferreira Bertolucci, Sonia Maria Dozzi Brucki, Ricardo Nitrini, Eliasz Engelhardt, Márcia Lorena Fagundes Chaves

**Affiliations:** 1 Universidade Federal de Pernambuco, Centro de Ciências Médicas, Área Acadêmica de Neuropsiquiatria, Recife PE, Brasil.; 2 Instituto de Medicina Integral Prof. Fernando Figueira, Recife PE, Brasil.; 3 Universidade de São Paulo, Faculdade de Medicina, Departamento de Neurologia, Grupo de Neurologia Cognitiva e do Comportamento, São Paulo SP, Brasil.; 4 Faculdade de Medicina da Universidade Federal do Ceará. Fortaleza, Brasil.; 5 Universidade Federal do Maranhão, Centro de Ciências da Saúde, Departamento de Medicina I, São Luís MA, Brasil.; 6 Instituto D’Or de Pesquisa e Ensino, Rio de Janeiro RJ, Brasil.; 7 Universidade de São Paulo, Faculdade de Medicina, Divisão de Geriatria, São Paulo SP, Brasil.; 8 Pontifícia Universidade do Rio Grande do Sul, Escola de Medicina, Serviço de Neurologia, Porto Alegre RS, Brasil.; 9 Pontifícia Universidade do Rio Grande do Sul, Instituto do Cérebro do Rio Grande do Sul, Porto Alegre RS, Brasil.; 10 Pontifícia Universidade do Rio Grande do Sul, Programa de Pós-Graduação em Gerontologia Biomédica, Porto Alegre RS, Brasil.; 11 Universidade Estadual de Campinas, Faculdade de Ciências Médicas, Departamento de Neurologia, Campinas SP, Brasil.; 12 Hospital Geral de Fortaleza, Serviço de Neurologia, Fortaleza CE, Brasil.; 13 Universidade de Fortaleza, Fortaleza CE, Brasil.; 14 Universidade Federal de Minas Gerais, Departamento de Clínica Médica, Belo Horizonte MG, Brasil.; 15 Universidade Federal de São Carlos, Centro de Ciências Biológicas e da Saúde, Departamento de Medicina, São Carlos SP, Brasil.; 16 Universidade Federal de São Paulo, Escola Paulista de Medicina, Departamento de Neurologia e Neurocirurgia, São Paulo SP, Brasil.; 17 Universidade Federal do Rio de Janeiro, Instituto de Neurologia Deolindo Couto e Instituto de Psiquiatria, Rio de Janeiro RJ, Brasil.; 18 Hospital de Clínicas de Porto Alegre, Serviço de Neurologia, Porto Alegre RS, Brasil.; 19 Universidade Federal do Rio Grande do Sul, Faculdade de Medicina, Departamento de Medicina Interna, Porto Alegre RS, Brasil.

**Keywords:** Dementia, Vascular, Cognitive Dysfunction, Cerebral Infarction, Stroke, Demência Vascular, Disfunção Cognitiva, Infarto Cerebral, Acidente Vascular Cerebral

## Abstract

Since the publication of the latest recommendations for the diagnosis and treatment of Vascular Dementia by the Brazilian Academy of Neurology in 2011, significant advances on the terminology and diagnostic criteria have been made. This manuscript is the result of a consensus among experts appointed by the Scientific Department of Cognitive Neurology and Aging of the Brazilian Academy of Neurology (2020-2022). We aimed to update practical recommendations for the identification, classification, and diagnosis of Vascular Cognitive Impairment (VCI). Searches were performed in the MEDLINE, Scopus, Scielo, and LILACS databases. This guideline provides a comprehensive review and then synthesizes the main practical guidelines for the diagnosis of VCI not only for neurologists but also for other professionals involved in the assessment and care of patients with VCI, considering the different levels of health care (primary, secondary and tertiary) in Brazil.

## RESUMO

Desde a publicação das últimas recomendações para o diagnóstico e tratamento da Demência Vascular pela Academia Brasileira de Neurologia em 2011, avanços significativos ocorreram na terminologia e critérios diagnósticos. O presente manuscrito é resultado do consenso entre especialistas indicados pelo Departamento Científico de Neurologia Cognitiva e do Envelhecimento da Academia Brasileira de Neurologia (2020-2022). O objetivo foi atualizar as recomendações práticas para a identificação, classificação e diagnóstico do Comprometimento Cognitivo Vascular (CCV). As buscas foram realizadas nas plataformas MEDLINE, Scopus, Scielo e LILACS. As recomendações buscam fornecer uma ampla revisão sobre o tema, então sintetizar as evidências para o diagnóstico do CCV não apenas para neurologistas, mas também para outros profissionais de saúde envolvidos na avaliação e nos cuidados ao paciente com CCV, considerando as diferentes realidades dos níveis de atenção à saúde (primário, secundário e terciário) no Brasil.

## INTRODUCTION

Vascular Cognitive Impairment (VCI) is the term used to include the entire spectrum of changes in cognition directly or indirectly related to cerebrovascular disease^1^. It is a construct proposed by Vladimir Hachinski (1994) to describe cases associated with “cerebrovascular disease” (CVD) ^(^ ^2^ and partially replace the concept of “Vascular Dementia” (VD) proposed by Carlo Loeb^3^. The term VCI identifies and includes all forms and severity levels of cognitive impairment, constituting a continuum of clinical and pathological presentations, from an asymptomatic stage (the “brain-at-risk) to dementia VD, including an intermediate stage of clinical deficits that do not reach the dementia criteria, initially called the “pre-dementia” stage^2^ ^),(^ ^4^, which was named “Vascular Cognitive Impairment Non-Dementia” (VCIND) ^(^ ^5^ ^),(^ ^6^ or “Vascular Mild Cognitive Impairment” (VMCI) ^(^ ^7^ ^)-(^ ^9^. Thus, the symptomatic spectrum of the condition comprises VCIND/VMCI and VCI (or VD).

Since the publication of the latest recommendations for the diagnosis and treatment of Vascular Dementia by the Brazilian Academy of Neurology in 2011^10^ ^)-(^ ^12^, significant advances on the terminology and diagnostic criteria has been made^1^ ^),(^ ^9^ ^)-(^ ^15^. New markers of structural and functional neuroimaging allowed the understanding of the heterogeneity of clinical presentations of VCI, including the compensation mechanisms of neural networks^16^. We also highlight contributions from Brazilian and Latin American groups to the area, which will be highlighted throughout the text.

This guideline seeks to provide a comprehensive review and then synthesize the main practical guidelines for the diagnosis of VCI not only for neurologists but also for other professionals involved in the assessment and care of patients with VCI, considering the reality different levels of health care (primary, secondary and tertiary) in Brazil and Latin America.

## METHODS

This manuscript is the result of a consensus among experts appointed by the Scientific Department of Cognitive Neurology and Aging of the Brazilian Academy of Neurology (2020-2022). We aimed to update practical recommendations for the identification, classification, and diagnosis of VCI. There are currently several updated guidelines and consensuses on the topic, many of which are cited and discussed throughout the text, so it was not our objective to carry out a new systematic review or exhaustive classification of the evidence.

Searches were performed in the MEDLINE, Scopus, Scielo, and LILACS databases until June 2021, using the descriptors “vascular cognitive impairment” or “vascular dementia”. We selected mostly articles published in the last 10 years, but older relevant publications were also included. Articles in English, Portuguese, and Spanish were reviewed. We also revised the reference list of the articles for relevant additional references. Review articles were also included when applicable.

## EPIDEMIOLOGY AND RISK FACTORS

The prevalence of VCI is too complex to estimate, because of geographic factors that imply in very heterogeneous societies, the huge variation in the criteria used for diagnosis, different complementary methods used in the investigation, or the scarcity of studies, especially in Brazil and in low- and middle-income countries^17^ ^),(^ ^18^. Despite the lack of standardized diagnostic criteria, making it difficult to determine its prevalence and risk factors (RF), VD is accepted as the second leading cause of dementia in the elderly, ranging from 8-45% of cases^19^ ^),(^ ^20^.

Studies with post-stroke patients have detected VCIND in 24 to 70% of cases^21^ ^),(^ ^22^. Considering that VD usually affects up to a third of individuals who have suffered a stroke, it is observed that the VCIND segment has a higher prevalence than dementia conditions^23^. A Brazilian study evaluated 172 patients one year after an ischemic stroke and found that 12.2% of cases met the criteria for probable VD^24^. Another study estimated that approximately 5% of individuals over 65 years of age had VCI, with 2.4% in the VCI-ND stage and 1.5% in the DV stage^6^. Similarly, review studies showed that the prevalence of VMCI ranged between 21 and 30%, affecting 24-75% in cases with diagnosed stroke and 4-19% in those in which stroke had not been reported^25^. The high prevalence of VCI-ND/VMCI highlights the importance of this etiology of dementia, especially considering that early diagnosis and treatment of RF for VCI can prevent, stabilize, or prevent the development of VD^19^ ^),(^ ^26^ ^)-(^ ^28^. A clinicopathological study by the Biobank for Aging Studies of the University of São Paulo described a prevalence of DV of 35%, considering only the presence of chronic infarcts for the neuropathological diagnosis, increasing to 49% when the presence of moderate to severe small vessel disease was included in the neuropathological criteria for VD^29^. It should be remembered that mixed forms of vascular pathology with neurodegenerative disease [e.g., VCI + Alzheimer’s disease (AD)] are also included in the VCI construct, with important participation in the total prevalence of VCI^19^ ^),(^ ^30^ ^),(^ ^31^. The possibility that mixed forms have their evolution attenuated and/or delayed through preventive measures is another aspect of great importance^2^ ^),(^ ^19^ ^),(^ ^3^.

The RFs for VCI are diverse. They are classically divided into sociodemographic, clinical characteristics, neuroimaging aspects, and VCI characteristics. Non-modifiable RFs include advanced age, gender, ethnicity, and genetic aspects (CADASIL, CARASIL, VLDL-R, APOE ε-4, HERNS, FABRY, among others). Classic metabolic and cardiovascular RFs are hypertension, diabetes, dyslipidemia, atrial fibrillation, previous stroke, metabolic syndrome, obesity, glucose intolerance, elevated homocysteine, carotid stenosis, and hyperuricemia. Toxic RFs include alcoholism, smoking, and other causes such as low education, sedentary lifestyle, inadequate diet, sleep apnea, and depression^33^.

## MECHANISMS AND PATHOPHYSIOLOGY

Cerebral vascular injuries comprise the ischemic (infarcts, microinfarcts, lacunae, white matter hyperintensities, enlarged perivascular spaces) and hemorrhagic lesions (hemorrhagic infarcts, cerebral hemorrhages, and microhemorrhages), which present in a variable way, with no single neuropathological lesion characterizing VCI. In addition, there are no widely accepted criteria in relation to the location and number of lesions necessary for the neuropathological diagnosis of VCI^34^ ^)-(^ ^37^. Several neuropathologic events seem to contribute to the occurrence of VCI, including loss of white matter integrity with consequent disconnection between strategic areas for cognitive networks^16^, changes in the coagulation cascade^38^ and oligodendrocytes^39^, and changes in endothelial cells with alterations in cerebral blood perfusion^40^.

The presence of lesions on neuroimaging must be interpreted considering the clinical context. To cause clinical symptoms, several basic characteristics must be met, such as extension, location and number of lesions. In addition, other factors can influence the clinical outcome of injuries, such as diaschisis, compensation mechanisms, and cognitive reserve^41^ ^)-(^ ^43^.

The parameters for VCI-ND and DV regarding location, extension, and the number of lesions were previously examined by several authors. Lesions located in limbic-paralimbic regions, heteromodal associative areas, certain subcortical structures, or in their connections tend to produce especially relevant pictures of VCI^44^ ^),(^ ^45^. Thus, lesions in the following areas are related to clinical symptoms: the anterior cerebral artery (affecting the prefrontal region), the middle cerebral artery (associative areas of the parietal lobe, parietotemporal, temporo-occipital), the posterior cerebral artery (inferotemporal region), hippocampus, and thalamus nuclei [anterior, medial dorsal]) ^(^ ^45^ ^)-(^ ^47^. As for the white matter, which is partly made up of long intra-hemispheric bundles, the frontosubcortical pathways (fronto-striatum-pale-thalamus-frontal circuits) underlying the executive function should be highlighted. Damage to these tracts is frequent in cases of VCI, even in theearly stages^48^. It should also be remembered that, regardless of their location, white matter lesions in any location compromise the frontal function^49^.

However, the extent and number of detectable lesions have been less studied. The most recent criteria for VCI pointed to the need for a lower vascular load (fewer number of lesions) for the diagnosis of non-dementia presentations^1^ ^),(^ ^9^ ^),(^ ^14^ ^),(^ ^50^. It is important to remember that white matter hyperintensities (WMH) are not homogeneously constituted and may present with different degrees of tissue alteration, with varied rarefaction. This aspect has been described in histopathology and diffusion tensor studies^51^ ^),(^ ^52^.

**Figure 1:**
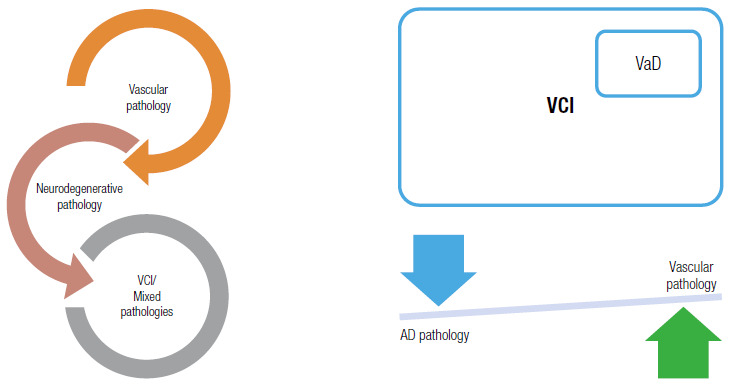
A and B. Relationship between vascular cognitive impairment and dementia, adapted^99^. VCI refers to any degree of cognitive decline related to cerebrovascular pathology, from the pre-clinical stages (brain at risk), through VMCI and dementia. VCI can be an isolated cause of cognitive decline or, to some degree, coexist with neurodegenerative pathology such as AD. The term Vascular Dementia refers to the subgroup of patients whose cognitive decline is mostly cerebrovascular in nature. VCI: vascular cognitive impairment; VaD: vascular dementia.

In pathologic studies, the definition of a neuropathologic threshold to consider the lesion as a cause of cognitive alteration in VCI is a difficult task^53^ ^),(^ ^54^. The same can be said about VCI as a whole. Furthermore, evidence from the recent decades indicates that isolated DV is much less prevalent than mixed VCI, a product of degenerative AD type and cerebrovascular lesions^55^. VCI Changes at any stage can be associated with neurodegenerative disorders, such as AD, constituting mixed pictures (such as VD+AD) ^(^ ^23^ ^),(^ ^56^, as well as other conditions (Frontotemporal lobar degeneration, Dementias with Lewy’s Body) (Figure 1 A and B) ^(^ ^1^.

## THE VASCULAR COGNITIVE IMPAIRMENT SPECTRUM

### Brain-at-risk

White-matter changes - especially symmetrical bilateral punctiform lesions, located in periventricular and deep subcortical regions - are commonly found in healthy elderly subjects^57^. Although often detected in late-life, WMH on T2 and FLAIR (*fluid attenuated inversion recovery*) magnetic resonance imaging (MRI) are not inherent features of normal brain aging^58^. In fact, their occurrence is strongly associated with the presence of vascular-related RF, such as metabolic diseases, smoking, among others ^59^.

**Figure 2:**
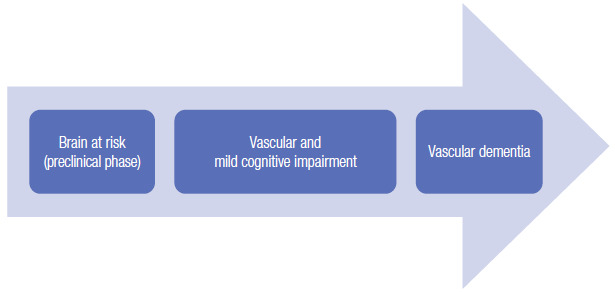
The spectrum of vascular cognitive impairment.

In addition, according to meta-analyses, extensive WMH burden conferred a 73-84% increased risk of incident dementia^59^ ^),(^ ^60^. Hence, the observation that these neuroimaging findings precede the onset of cognitive and behavioral abnormalities suggest that, similarly to AD^61^, a *preclinical stage* may exist in VCI (Figure 2).

With the high prevalence of cerebrovascular disease in older population, determining the odds of cognitive decline attributed to individual or combined biomarkers, including the lesion type at neuroimaging (WMH, lacunes, microbleeds, perivascular space dilations, amyloid angiopathy etc.), the lesion load, the speed of infarct recurrence and the “allostatic load” (which refers to the cumulative effects of multiple vascular RF) is needed. The idea that broadly available therapeutic interventions may effectively participate in the primary prevention of symptomatic VCI emphasizes the importance of more studies aiming at the characterization of this stage^62^ ^),(^ ^63^.

### Vascular Cognitive Impairment No-Dementia / Vascular Mild Cognitive Impairment (VCIND / VMCI) 

Within the VCI spectrum, the earliest symptomatic phase, in which cognitive impairment does not fulfill dementia criteria, has been referred to as VCIND^5^ ^),(^ ^6^ or VMCI^7^ ^)-(^ ^9^. Moreover, the 5^th^ edition of the Diagnostic and Statistical Manual of Mental Disorders (DSM-5) suggested a novel nomenclature, which was endorsed by the 11^th^ edition of the International Classification of Diseases (ICD-11) ^(^ ^15^ ^),(^ ^64^. In these publications, Vascular Dementia (VD) was identified as “Major Neurocognitive Disorder”, whereas VCIND / VMCI was renamed as “Mild Neurocognitive Disorder” ^(^ ^15^ ^),(^ ^64^.

These conditions are associated with substantial risk of cognitive worsening and progression to dementia^65^. Longitudinal studies reported that 22 to 58% of subjects diagnosed as VCIND progressed to VD after 2-7 years of follow-up^66^ ^)-(^ ^69^. In contrast, cognitive recovery was detected in 8-45% of the cases, while 38-74% remained cognitively stable. Among those in the latest group, 30-34% presented subtle cognitive decline, which was not sufficient for a transition from VCIND to VD^66^ ^)-(^ ^69^.

Furthermore, risk of progression towards dementia may vary across individuals classified as VCIND. According to the DSM-5, magnitude of impairment in neuropsychological testing in this diagnostic group corresponds to performances between 1 to 2 standard deviations (SD) from mean normative values^15^. A longitudinal study reported that three levels of severity could be distinguished within this category: mild VCIND (cognitive deficits up to 1 SD from normative data), moderate VCIND (cognitive impairments of 1.5 SD from normative scores) and severe VCIND (performances up to 2 SD from expected scores, considering age and education). It has been indicated that higher severity of cognitive abnormalities in VCIND correlated with increased odds of transition to dementia^70^.

Additionally, the number of vascular-related RF may impact on cognitive performances, with those with more elevated vascular burden presenting poorer scores in neuropsychological tasks^71^ ^),(^ ^72^. Table 1 depicts the most relevant diagnostic criteria for VCIND.

**Table 1:** Diagnostic Criteria for Vascular Cognitive Impairment, No Dementia (VCIND).

Criteria	Description
Zhao et al., 2010^49^	**VCIND**
	Cognitive impairment in ≥ 1 domain.
	ADLs maintained.
	Does not meet accepted criteria for the diagnosis of dementia.
**VCCID** Gorelick et al., 2011^9^	**VMCI**
	Includes the 4 proposed subtypes for MCI: amnesic, amnesic + other domains, non-amnestic single domain, and non-amnestic multiple domains.
	The VMCI classification must be based on cognitive tests and at least 4 cognitive domains must be assessed: executive/attention, memory, language, and visuospatial functions.
	Classification should be based on presumed decline in cognitive function compared to a previous baseline and impairment in ≥ 1 cognitive domain.
	IADLs can be normal or mildly compromised (regardless of motor/sensory symptoms).
**VASCOG** Sachdev et al., 2014^14^	**Mild VCD**
	Evidence of significant cognitive decline in > 1 domain compared to previous level of performance.
	Cognitive impairment between 1 and 2 SD below the mean (or between the 3^rd^ and 16^th^ percentile) (compared to individuals of similar age, sex, education and social-cultural profile).
	Fronto-executive deficiencies are more likely to be present.
	Preserved IADLs (the individual, although still independent, performs tasks with greater effort and uses compensation strategies).
**VICCCS** Skrobot et al., 2018^1^	**Mild VCI**
	Impairment in ≥ 1 cognitive domain.
	BADLs or IADLs maintained or with mild impairment (regardless of motor/sensory symptoms).
**ABN 2021 Consensus Proposal**	**CCVND**
	Cognitive impairment in ≥ 1 cognitive domain.
	Cognitive impairment between 1 and 2 SD below the mean (or between the 3^rd^ and 16^th^ percentile).
	BADLs maintained (regardless of motor/sensory symptoms).
	Preserved IADLs (although with greater effort + compensation strategies).

VCCID: Vascular Contributions to Cognitive Impairment and Dementia; VASCOG: International Society for Vascular Behavioral and Cognitive Disorders; VICCCS: Vascular Impairment of Cognition Classification Consensus Study; VCIND: vascular cognitive impairment, no dementia; VMCI: vascular mild cognitive impairment; ADL: activities of daily living; BADLs: basic activities of daily living; IADLs: instrumental activities of daily living; MCI: mild cognitive impairment; mild VCD: mild vascular cognitive disorder; SD: standard deviations.

### Vascular Dementia (VD) and its classification

Current diagnostic criteria for VD require the occurrence of significant impairment in at least one cognitive domain (although more domains may be affected), and severe functional disability, including difficulties to perform instrumental or basic activities of daily living^1^ ^),(^ ^9^ ^),(^ ^14^. Noteworthy, defining thresholds to characterize “severe functional disability” might be challenging, since an array of skills are implicated in one’s capacity to exert everyday life activities, such as cognitive, behavioral, sensorial, and motor factors. Table 2 summarizes the main diagnostic criteria for VD.

**Table 2:** Diagnostic Criteria for Vascular Cognitive Impairment - Dementia (VD)

Criteria	Description
**VCCID** Gorelick et al., 2011^9^	**VD**
	The diagnosis of VD should be based on a presumption of decline in cognitive function compared to a previous baseline and impairment in ≥ 2 cognitive domains sufficient to affect activities of daily living.
	The diagnosis of VD must be based on cognitive tests, and at least 4 cognitive domains must be assessed: executive/attention, memory, language, and visuospatial functions.
	Deficits in ADLs must be independent of the motor/sensory sequelae of the vascular event.
**VASCOG** Sachdev et al., 2014^14^	**Dementia, major VCD**
	Evidence of significant cognitive decline in > 1 domain compared to previous level of performance.
	Cognitive impairment ≥ 2 SD below the average (or below the 3^rd^ percentile) (compared to individuals of similar age, sex, education and socio-cultural profile)
	Fronto-executive deficiencies are more likely to be present.
	Enough to interfere with independence (at least requires help with IADLs, e.g., complex tasks such as managing finances or medications).
**VICCCS** Skrobot et al., 2018^1^	**Major VCI, VD**
	Impairment in ≥ 1 cognitive domain.
	Significant impairment of IADLs or ABVDs (regardless of motor/sensory symptoms).
**ABN 2021 Consensus Proposal**	**Major VCI, VD**
	Cognitive impairment in ≥ 1 cognitive domain.
	Cognitive impairment ≥ 2 SD below the mean (or below the 3^rd^ percentile).
	Significant impairment of IADLs or ADLs (regardless of motor/sensory symptoms).

VCCID: Vascular Contributions to Cognitive Impairment and Dementia; VASCOG: International Society for Vascular Behavioral and Cognitive Disorders; VICCCS: Vascular Impairment of Cognition Classification Consensus Study; VD: Vascular Dementia; major VCD: major vascular cognitive disorder; VCI: vascular cognitive impairment; ADL: activities of daily living; BADLs: basic activities of daily living; IADLs: instrumental activities of daily living; SD: standard deviations.

**Figure 3:**
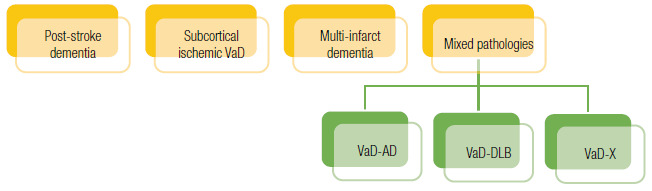
Classification of forms of vascular dementia according to the VICCCS (*Vascular Impairment of Cognition Classification Consensus Study*) (1). Each form will be further stratified into possible and probable (Table 3). Mixed forms can occur in all of the above syndromes, and the suspected neurodegenerative syndrome should be detailed (VD-AD and VD-LBD were used as examples, with X denoting other possible associations).

Once diagnosed as VD, patients should be investigated for the underlying pathology (Table 3, Figure 3), and for the level of certainty of the vascular etiology - check the next section for detailed information about this theme. Objective 6-month temporal relationship between a cerebrovascular ictus and the onset of cognitive abnormalities is only mandatory for the characterization of *post-stroke dementia*.

**Table 3:** Main forms of major VCI / Vascular Dementia, adapted from^1^.

Classification	Description
Post-stroke dementia	Presence of new, sudden or subacute cognitive deficit up to 6 months after ischemic or hemorrhagic stroke. It may be due to different cerebrovascular patterns (e.g., multiple cortico-subcortical infarctions, strategic lesions, subcortical vascular dementia, etc.). The temporal relationship between the vascular event and cognitive decline differentiates this form from VD.
Mixed dementias	Broad term that encompasses cognitive decline phenotypes combined between VCI and neurodegenerative diseases (e.g. VCI-AD, VCI-LBD etc). It is recommended to specify which underlying pathology is suspected, avoiding the less specific term “mixed dementia.”
Subcortical ischemic vascular dementia	Small vessel cerebrovascular disease is the main cause in this group, mainly due to lacunar infarcts and white matter lesions. It encompasses the phenotypes described as Binswanger’s Disease and the lacunar state.
Cortical multiple infarct dementia	A group characterized by the presence of multiple cortical infarcts and their likely contribution to dementia.
Level of certainty	Possible - more appropriate term if neuroimaging is unavailable. Probable - in the presence of compatible CT or MR. MR is the method of choice.

VCI: vascular cognitive impairment; VD: vascular dementia; AD: Alzheimer’s disease; LBD: Lewy Body dementia; CT: computer tomography; MR: magnetic resonance.

VaD: vascular dementia; AD: Alzheimer’s disease; LBD: Lewy Body Dementia.

Other syndromes related to VD have been previously described in the literature^73^ ^),(^ ^74^. Among those, small vessel disease associated with Cerebral Amyloid Angiopathy (CAA) deserve to be commented. In addition to its relationship with cerebral hemorrhage, CAA has been linked to AD pathology in post-mortem analyses^75^. Cognitive dysfunction may occur in these cases, even without evident brain hemorrhage. Some neuroimaging features associated with CAA include lobar microbleeds, lobar intraparenchymal hemorrhage, cortical superficial siderosis, WMH, convexity subarachnoid hemorrhage, and dilated perivascular spaces^74^.

Among genetic syndromes, Cerebral Autosomal Dominant Arteriopathy with Subcortical Infarcts and Leukoencephalopathy (CADASIL), caused by mutations in the *NOTCH3* gene, ought to be outlined. This condition induces deposition of granular osmiophilic material in the walls of vascular smooth muscle cells. Clinical manifestations encompass early-onset VD, with remarkable decrease in cognitive speed, executive dysfunction, and attentional deficits, as well as depression, headache and positive family history. Brain MRI often evidences substantial white-matter damage, as a result of subcortical infarcts, and affected temporal poles. A similar phenomenon may be observed in autosomal recessive mutations in the *HTRA1* gene, which causes Cerebral Autosomal Recessive Arteriopathy with Subcortical Infarcts and Leukoencephalopathy (Cerebral autosomal recessive arteriopathy with subcortical infarcts and leukoencephalopathy - CARASIL). Awareness should be raised to this diagnosis when alopecia and spondylosis are detected along with the typical signs and symptoms of CADASIL^74^.

## CLINICAL MANIFESTATIONS AND DIAGNOSTIC WORKUP OF VCI/DEMENTIA

The clinical manifestations of VCI/dementia include cognitive impairment, functional decline, neuropsychiatric symptoms, neurological manifestations and autonomic dysfunction, in variable proportions and associations, according to the type, location, number and extent of the lesions^10^ ^),(^ ^11^.

**Figure 4:**
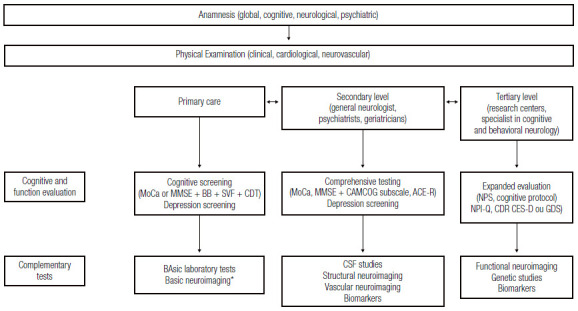
Flowchart proposed for the assessment and investigation of patients with suspected VCI / VD. *Basic neuroimaging refers at least to Computed Tomography of the Skull. MoCa: Montreal cognitive assessment; MMSE: Mini-Mental State Examination; BB: Brief Battery of Cognitive Screening; SVF: semantic verbal fluency; TDR: clock design test; CAMCOG: Cambridge Cognitive Examination; ACE-R: Addenbrooke’s Cognitive Examination-Revised; NPS: neuropsychological assessment; NPI-Q: Neuropsychiatric Inventory Questionnaire; CDR: clinical dementia rating; CES-D: Center for Epidemiologic Studies - Depression; GDS: Geriatric Depression Scale.

A detailed protocol must be followed to establish diagnosis, including several steps, i.e., clinical history (clinical, cognitive, neurologic, psychiatric), physical examination (clinical-cardiological, neurologic), neuropsychological assessment (screening, comprehensive assessment), functional assessment, neuropsychiatric evaluation, and diagnostic exams (neuroimaging, laboratory testing, among others) ^(^ ^9^ ^),(^ ^23^ ^),(^ ^32^ (Figure 4).

### Which components of the history are essential in evaluating patients with suspected VCI and dementia? ^(^ ^76^

The patient’s history is essential for characterizing cognitive deficits, generating a differential diagnosis, and determining the cause of dementia. The best way to do this is to identify medical, neurologic and psychiatric symptoms as clues to the probable cause of the cognitive changes, establishing the order of appearance, the severity and the associated features. Ideally in VD the loss of function should be temporally correlated with cerebrovascular events. A reliable relative/informant plays an important role in providing information since cognitive dysfunction may impair the patient’s ability to report accurately.

VD should be suspected in any patient presenting cerebrovascular RF, even if the neurologic examination does not suggest stroke. A stepwise deterioration may be observed. It may be present in patients with silent stroke, in those with several small strokes, or in those with severe diffuse subcortical cerebrovascular disease.

### Which methods clinicians should use to detect VCI/VD? 

At first, the indiscriminate assessment of elderly individuals for dementia is not recommended^77^ ^),(^ ^78^. In the evaluation of older patients for dementia, clinicians should use a standardized screening tool, along with a brief patient history obtained from a reliable informant (a person who is directly in contact with the patient).

### Screening tools

The screening instrument must be easy to use, highly sensitive, widely available and supported by populational data^76^. The Mini-Mental State Examination (MMSE) is widely used and contributes to the diagnosis of dementia in low prevalence settings. However, it should not be used in isolation to confirm or exclude the disease^79^. To improve diagnostic accuracy in low educated populations, we suggest the association of Brief Cognitive Battery^80^ ^)-(^ ^82^, which includes an interference with a semantic verbal fluency (animals) and the clock drawing test (CDT). Other options are the Mini-Cog^83^ and the Montreal Cognitive Assessment (MoCA) ^(^ ^84^.

The Mini-Cog has the benefit of brevity and the MoCA has the best sensitivity but lower specificity^85^. The MoCA was originally developed for the detection of MCI and may be difficult for people with moderate or advanced dementia, as well as for populations with low educational backgrounds. For those whose previous cognitive function was measured with the MMSE, there is a tool that links the MoCA scores to the corresponding MMSE ones^86^. A meta-analysis carried out by the Cochrane showed sensitivity of 0.76 and specificity of 0.73 of the Mini-Cog for the diagnosis of dementia in general^87^. For the diagnosis of dementia in the primary care settings, the number of studies evaluating the accuracy of the Mini-Cog was limited.

Despite the large amount of short cognitive screening tools, few are valid for patients with suspected VCI. A systematic review on screening tests for the identification of VCI^88^, the MoCA, the MMSE, the Brief Memory and Executive Test - BMET - and different versions of the Clock Design Test were the most widely validated instruments. Based on available evidence, the authors concluded that the MoCA was the most accurate and reliable instrument, however this finding still needs further validation in our population. The BMET has already been adapted for Brazilian individuals^89^.

### Cognitive assessment tests in the context of VCI

Cognitive screening tests used in the assessment of AD, particularly the MMSE, are not ideal for vascular cognitive impairment. Those including the assessment of frontal, executive, and subcortical functions are preferred. Modifications of tests originally developed for AD, such as the Vascular Version of the Alzheimer’s Disease Assessment Scale-Cognitive Subscale (VADAS-Cog), may be useful^90^. Some of them may be able to differentiate AD from VCI, but even with the use of biomarkers to eliminate the presence of AD pathology (e.g., a negative amyloid PET), some overlap may persist between the cognitive changes of vascular cognitive impairment and AD^91^. Validation data exist for the MoCA^92^ ^),(^ ^93^ and for the Addenbrooke Cognitive Exam Revised Version^94^, both already validated for the Brazilian population^95^ ^)-(^ ^97^. There are some suggestions for protocols of cognitive assessment for the detection of VCI/VD that have been published in the last two decades and are summarized in Suplementary Material.

In Brazil, a previous review on VD was published by the Scientific Department of Cognitive Neurology from the Brazilian Academy of Neurology (ABN) ^(^ ^10^. In this document, the expert panel emphasized that the pattern of cognitive changes was highly variable, requiring sufficient sensitivity from the neuropsychological protocols to detect a wide range of domains, mainly executive function. The selected tests must meet criteria of frequency and validity, be freely available, and be well known and sensitive to detect cognitive decline. The protocols must be broad, easy to administer and relatively brief^32^. The recommendations included a brief screening protocol for VCI/VD, consisting of the MMSE, the semantic verbal fluency test (animals) and the CDT^10^. The authors also included a broader and time-consuming protocol composed of a wider range of tests (Appendix). This version included the Cambridge Cognitive Examination (CAMCOG) scale with the global and subscale scores, which has been adapted and validated for the Brazilian population^98^ ^),(^ ^99^.

The National Institute for Neurological Disorders and Stroke (NINDS) and the Canadian Stroke Network established a working group to define criteria for VCI^23^. In this document, the Neuropsychological Working Group proposed three separate protocols which were recommended for multicenter investigations with VCI patients, one requiring at least 60 minutes, a second of 30 minutes and a third of five minutes (Suplementary Material). The longer one, 60 minutes, was developed for use in studies requiring an analysis of cognitive skills by domain, thus the protocol contained recommended tests in four domains: executive/activation, language, visuospatial and memory. In addition, tests were selected to examine changes in behavior and mood. The other two protocols were selected from within the 60-minute protocol to be used as a clinical screening tool for suspected VCI patients. The 5-minute protocol was projected for potential use by primary care physicians, nurses, and other health care professionals. The 5-minute protocol was also designed for large epidemiological studies or clinical trials where sensitivity and ease of administration are especially important. In addition, once validated the 5-minute protocol was also designed to be administered over the phone. Most of the tests included in these protocols are available in Brazil, especially the 5-minute version that is sourced from MoCA.

Based on all the above data, this panel recommends the use of screening/cognitive assessment tests for the detection of VCI according to the level of health care in which the patient is inserted (Table 4).

**Table 4:** Cognitive screening recommendation on vascular cognitive impairment stratified by health care levels.

Health level	Cognitive screening recommendation for CCV
Primary attention	General practitioners and professionals from the Family Health Strategy can use screening instruments such as MoCA, or alternatively the MMSE associated with the clock drawing test and semantic verbal fluency (animals) and screening for depressive symptoms.
Secondary Care	Specialist physicians (neurologists, geriatricians, and psychiatrists) who receive patients referred from primary care can use a broader protocol that includes global function tests (MoCA or MMSE) associated with CAMCOG subscales, or Addenbrooke battery (ACE-R) and screening for depressive symptoms.
Tertiary care	Specialist physicians at referral centers can use expanded assessment including, in addition to a cognitive protocol, the assessment of neuropsychiatric symptoms (NPI-Q), assessment of severity of dementia (CDR), and screening for depressive symptoms (CES-D or GDS).

### Assessment of functionality

The use of the Clinical Dementia Rating scale (CDR) presents some difficulties for cases of vascular nature, although the scale has been validated for such cases in the Brazilian settings^100^. CDR is strongly based on memory impairment. However, other domains, such as executive function, are especially important in vascular cases.

A study of subcortical VCI (70% of cases with CDR 0.5) showed, in cases of moderate and severe subcortical lesions, that the sum of the “functional” boxes (judgment/problem solving, community affairs, home/hobbies and personal care) (CDR FUNC) of the CDR presented correlation with the Pfeffer’s Functional Activities Questionnaire (FAQ), CLOX 2, working memory and abstraction^101^.

A study with the CDR scale defined mild VCI as a MCI status of VCI or a CDR 0.5 status with cardiovascular disease. Thus, for the assessment of daily life the information from caregivers is necessary, especially in the domains (“boxes”) of “community affairs”, “home and hobbies” and “personal care” ^(^ ^25^. Thus, the use of the CDR scale can be accepted, as long as the sum of the functional boxes is considered valid.

### Laboratory diagnosis

No laboratory test or biomarker is specific for VCI. Conversely, routine exams can assess comorbidities or RF for cognitive decline. Evaluation of blood count, serology, glucose levels, B12, thyroid, kidney, and liver function, is often required - as detailed in a specific article of the present consensus. A metabolic profile with cholesterol and triglycerides is also important. AD biomarkers (beta-amyloid, tau, and phospho-tau) in the CSF may play a role in selected cases when the presence of mixed pathology is questionable^102^. Other CSF measurements (proteins, electrophoresis) may help to differentiate inflammatory causes or suggest a blood-brain barrier dysfunction (e.g., vasculitis or demyelinating diseases), albeit they are not part of the routine assessment of suspected cases of VCI^103^.

### Neuroimaging diagnosis

Neuroimaging is critical to detecting CVD causing VCI. MRI is the best and most accurate technique to visualize lesions produced by large vessels (infarctions), lesions resulting from small vessel diseases (WMH, small subcortical infarcts, lacunae, enlarged perivascular spaces, cerebral microhemorrhages). Cerebral hemorrhages (lobar, deep) can be well visualized through computed tomography (CT) ^(^ ^25^.

Currently, there are no criteria to define the necessary load of vascular lesions detected by neuroimaging to confirm the presence of VMCI, lacking a cutoff point for such definition^104^. Macroscopic findings of conventional neuroimaging (FLAIR sequences, CT) often partially explain the clinical expression (phenotype) of VD and usually represent heterogeneous pathological alterations^52^. Thus, the presence of hidden aspects (“invisible changes”) could contribute to clinical expression, which can be verified by more advanced techniques, such as diffusion tensor imaging (DTI) or other techniques capable of identifying areas of “WM of normal appearance” ^(^ ^51^ ^),(^ ^105^. In addition, advanced MRI techniques with analysis of structural and functional brain connectivity can contribute to possible investigations of how changes in more complex brain networks can explain the diversity of clinical presentations in these conditions, even in cases with anatomical changes in similar conventional sequences^16^, which can optimize the anatomical-clinical correlation in the future.

A recent systematic review on VMCI (subcortical), analyzing studies that assessed lesions qualitatively (WM lesion extension) and semi quantitatively (number of lacunes), showed the presence of moderate to severe vascular lesion load^106^.

**Figure 5:**
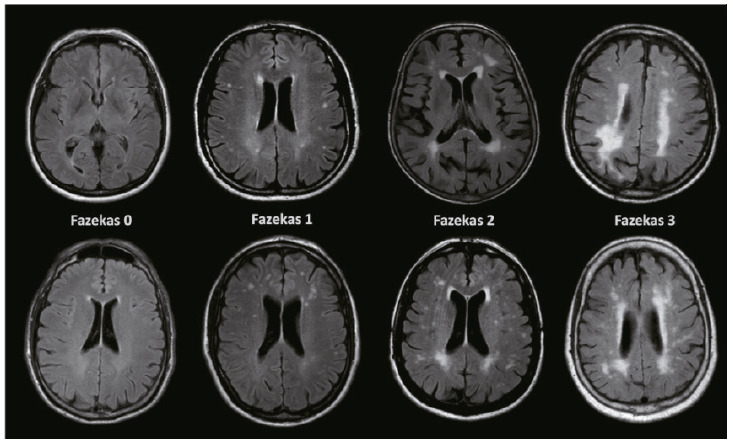
Evaluation of White Substance Hyperintensities according to the Fazekas visual scale^107^.

The application of visual scales can help the clinician in the differential diagnosis, in the anatomical-clinical correlation, especially with cognitive alterations, as well as in therapeutic monitoring^107^ (Table 5) (Figure 5). Conversely, the characterization of CVD through neuroimaging also usually contributes to the systemic treatment of the patient. The occurrence of WM lesions, either periventricular (PVWM) or diffuse (DWM), may exhibit differences in etiopathogenesis; for example, a combination of granular ependymitis (*ependymitis granularis*) and axonal demyelination may be related to PVWM^112^, whereas gliosis of the subependymal areas (subependymal gliosis) combined with chronic small vessel ischemia seems to contribute in more significant proportion to the occurrence of DWM^113^.

**Table 5:** Main visual scales in CVD.

Scale [reference]	Brain region [indication]	Method [scores]
Fazekas et al. ^(^ ^114^		Periventricular hyperintensities:
		0=absent
		1= “hoods” or thin coat
		2=smooth halo
	White matter hyperintensities	3=periventricular hyperintensities extending into deep white matter
	periventricular and deep	Deep white substance:
		0=absent
		1=point-like foci
		2=initial confluence
		3=large confluent areas
Modified Fazekas (LADIS) ^(^ ^115^	Deep white matter and subcortical lesions (DSWM)	0=absent
		1=[mild] punctiform lesions with maximum unit diameter below 10 mm and areas of clustered lesions smaller than 20 mm
		2=[moderate] single lesions between 10-20 mm in diameter, areas of clustered lesions greater than 20 mm in diameter, no more than connecting bridges between individual lesions 3=[severe] single lesions or confluent areas of hyperintensities 20 mm or more in diameter
Scheltens et al. ^(^ ^116^	PVH, DWMH, basal and infratentorial ganglia hyperintensities	PVH (0-6), WMH (0-24), basal and infratentorial ganglia (both 0-24)
		Total=0 to 84
ARWMC scale^117^	Hyperintensities of hemispheres and basal ganglia	White matter lesions:
		0 = absence
		1= focal lesions
		2=initial confluent lesions
		3= diffuse involvement of the entire region
		Basal ganglia:
		0=absence
		1=focal lesions (≥5 mm)
		2= >1 focal lesion
		3=confluent lesions
		Total = 0 to 30

MRI can offer advantages in observing PVWH and DWM through T2 and FLAIR sequences; conversely, both CT and MRI seem to identify cortical and lacunar infarcts in a similar way^106^. Evidence of hyperintensity, once nonspecific, is associated with CVD and AD pathology; the volume of WM alterations is regarded as a possible independent marker of beta-amyloid protein accumulation^114^.

The finding of lesions caused by multiple infarctions can direct clinical investigation towards the occurrence of coagulopathies, infectious and parasitic diseases, alcoholism, and heart failure^115^ ^),(^ ^116^. Perivascular disease, seen as microintensities at the cortico-subcortical junctions, has been independently associated with an increased risk for cognitive decline^117^. In Brazil and most countries of Latin America, the high occurrence of subcortical ischemic vascular disease is usually associated with late diagnosis of systemic hypertension, diabetes and dyslipidemia, as reported by the ELSA study^118^.

In conclusion, the evolution of the VCI concept from preclinical stages to VMCI and VD turns diagnostic assessment into a challenge for the multidisciplinary team. The patient with suspected VMCI should be evaluated from primary care by general practitioners, with complementary work up being carried out at the secondary and tertiary levels in a horizontal manner, according to the need for more advanced instruments, particularly advanced neuroimaging techniques.

## SUPPLEMENTARY MATERIAL

NINDS and the Canadian Stroke Network developed a 5-minute (12 points) subset of the MoCA to identify stroke patients who developed VCI^1^. As the MoCA itself, the total scores on this screening test were inversely correlated with age and positively correlated with education. The Oxford Cognitive Screen is a 15-minute test with fewer language elements than the MoCA, making it particularly useful in acute stroke patients who may have language deficits^2^. The LADIS neuropsychological battery was designed to assess the cognitive performance of a wide range of functions in a cohort of independently living individuals with age-related white matter changes (ARWMC) during a 3-year follow-up^3^. It is a short-comprehensive instrument that can be administered in a single visit. This battery was chosen based on the test familiarity and the instrument validity to assess cognitive decline in vascular disease patients. In addition to widely known and validated instruments (MMSE, Stroop Test, and Trail Tests), it included the VADAS-Cog^4^; detailed information on global and selective cognitive functioning and time-dependent tasks (Delayed Recall, Digit Symbol, Digit Extension, Maze, Digit Cancellation and Verbal Fluency) to complement the assessment of attention, mental processing speed and motor control were provided. These cognitive domains and executive function are possibly more affected by white matter alterations^5^. The authors concluded that the neuropsychological performance of the patients was significantly influenced by age and education, with a higher educational level being consistently associated with better performance on cognitive tasks. In contrast, older age was associated with difficulties in memory and executive functions.

Tuscany - Vascular Cognitive Impairment (VCI) is a multicenter, prospective, observational study aiming to evaluate predictors of transition from VCI (defined by the presence of moderate to severe WML) to dementia^6^. The neuropsychological test battery was designed specifically for MCI due to small vessel disease (MCI-SVD), enabling the automation and standardization of scores and a personalized cognitive profile. For the VCI-Tuscan neuropsychological battery, tests were selected among those recommended for VCI and the protocols proposed by the National Institute for Neurological Disorders and Stroke and the Canadian Stroke Network consensus conference on harmonization standards for VCI^7^. The definition of cognitive domains followed a confirmatory analysis of the dimensions assumed theoretically for the battery^8^, resulting in four cognitive clusters: memory (evaluated by four cognitive scores), attention and executive functions (five cognitive scores), language (two cognitive scores), and constructive praxis. Thus, the battery includes two tests of global cognitive functioning and another nine tests covering these cognitive domains.

**References**

1. Kennedy RE, Wadley VG, McClure LA, Letter AJ, Unverzagt FW, Crowe M, et al. Performance of the NINDS-CSN 5-minute protocol in a national population-based sample. J Int Neuropsychol Soc. 2014;20(8):856-67. doi:10.1017/S1355617714000733.

2. Mancuso M, Demeyere N, Abbruzzese L, Damora A, Varalta V, Pirrotta F, et al. Using the Oxford Cognitive Screen to Detect Cognitive Impairment in Stroke Patients: A Comparison with the Mini-Mental State Examination. Front Neurol. 2018;9:101. doi:10.3389/fneur.2018.00101.

3. Madureira S, Verdelho A, Ferro J, Basile AM, Chabriat H, Erkinjuntti T, et al. Development of a neuropsychological battery for the Leukoaraiosis and Disability in the Elderly Study (LADIS): experience and baseline data. Neuroepidemiology. 2006;27(2):101-16. doi:10.1159/000095381.

4. Ferris SH. General measures of cognition. Int Psychogeriatr. 2003;15(Suppl 1):215-7. doi:10.1017/S1041610203009220.

5. Mohs RC, Knopman D, Petersen RC, Ferris SH, Ernesto C, Grundman M, et al. Development of cognitive instruments for use in clinical trials of antidementia drugs: additions to the Alzheimer’s Disease Assessment Scale that broaden its scope. The Alzheimer’s Disease Cooperative Study. Alzheimer Dis Assoc Disord. 1997;11(Suppl 2):S13-21.

6. Poggesi A, Salvadori E, Pantoni L, Pracucci G, Cesari F, Chiti A, et al. Risk and Determinants of Dementia in Patients with Mild Cognitive Impairment and Brain Subcortical Vascular Changes: A Study of Clinical, Neuroimaging, and Biological Markers-The VMCI-Tuscany Study: Rationale, Design, and Methodology. Int J Alzheimers Dis. 2012;2012:608013. doi:10.1155/2012/608013.

7. Hachinski V, Iadecola C, Petersen RC, Breteler MM, Nyenhuis DL, Black SE, et al. National Institute of Neurological Disorders and Stroke-Canadian Stroke Network Vascular Cognitive Impairment Harmonization Standards. Stroke. 2006;37(9):2220-41. doi:10.1161/01.STR.0000237236.88823.47.

8. Salvadori E, Poggesi A, Pracucci G, Inzitari D, Pantoni L; VMCI-Tuscany Study Group. Development and psychometric properties of a neuropsychological battery for mild cognitive impairment with small vessel disease: the VMCI-Tuscany Study. J Alzheimers Dis. 2015;43(4):1313-23. doi:10.3233/JAD-141449.

**Table:** Characteristics of cognitive assessment protocols in different guidelines - cognitive tests included. References in the text.

LADIS Battery											
	MEEM	ADAS-Cog	COMMANDER					Trails A B	Stroop		
			Digit-symbol	Digit span	Maze	Cancellation	Verbal Fluency				
				WAIS-III		Digits					
Global Mental Functioning	+	+									
Orientation		+									
Memory		+		+							
Attention			+	+		+		+			
Language		+									
Constructional abilities		+									
Executive function			+				+	+	+		
Praxis		+									
Speed and motor control					+	+		+			
**MCIV-Tuscany Battery**											
	**MEEM**	**MoCA**	**Digit-symbol**	**Rey Test**	**Short story**	Rey-Osterrieth	**Verbal Fluency**	**Trails A B**	**Stroop**	**Visual search**	
				**(RAVL)**		**Copy Figure - Retrieval**					
				**immed - late**							
Global Mental Functioning	+	+									
Orientation											
Memory				+ +	+	+					
Attention + Executive function			+					+ +	+	+	
Language							+				
Constructional abilities						+					
**VCI-VICCCS criteria**											
	**MEEM**	**Boston Naming**	**Digit-symbol**	**Hopkins Verbal Learning Test**	**Simple reaction time with choice**	**Rey-Osterrieth**	**Verbal Fluency**	**NPI-Q**	**CES-D**		
						**Copy Figure- Retrieval**					
Global Mental Functioning	+										
Orientation											
Memory				+							
Attention			+			+					
Language		+					+				
Constructional abilities						+					
Executive function			+	+			+				
Speed and motor control					+						
Depressive symptoms									+		
Neuropsychiatric symptoms								+			
**Consensus Protocol A/ABN 2011**											
	**MEEM**	**Clock Drawing Test**					**Verbal Fluency**				
Global Mental Functioning	+										
Language							+				
Constructional abilities		+									
Executive function							+				
**Protocol B of consensus/ABN 2011**											
	**MEEM**	**CAMCOG (global and subscales)**	**Digit span**	**Trails A B**	**Boston Naming**	**Verbal Fluency**	**Word List - CERAD**	**CLOX**	**NPI**	**Cornell Depression Scale**	**CDR**
			**WAIS-III**								
Global Mental Functioning	+	+									
Orientation	+	+									
Memory							+				
Attention		+	+	+							
Language		+			+	+					
Constructional abilities		+									
Executive function			+	+		+		+			
Praxis		+									
Gnosia		+									
Abstract reasoning		+									
Depressive symptoms										+	
Neuropsychiatric symptoms									+		
Severity											+
**NINDS - Canadian Stroke Network Vascular Cognitive Impairment (Hachinski et al., 2006)**											
**30-minute Protocol**											
	**MEEM**	**Hopkins Verbal Learning test**	**Digit-symbol**	**Trails A B**	**Verbal Fluency**	**NPI-Q**	**CES-D**				
Global Mental Functioning	+										
Orientation											
Memory		+									
Attention			+	+							
Language					+						
Constructional abilities											
Executive function			+	+	+						
Speed and motor control											
Depressive symptoms							+				
Neuropsychiatric symptoms						+					
**5-minute Protocol - MoCA Subtests**											
	**Memory task with words**	**Orientation**	**Phonemic Verbal Fluency**								
Orientation		+									
Memory	+										
Attention	+		+								
Executive function			+								

## References

[1] Skrobot OA, Black SE, Chen C, DeCarli C, Erkinjuntti T, Ford GA (2018). Progress toward standardized diagnosis of vascular cognitive impairment: Guidelines from the Vascular Impairment of Cognition Classification Consensus Study. Alzheimers Dement.

[2] Hachinski V (1994). Vascular dementia a radical redefinition. Dementia.

[3] Loeb C (1985). Handbook of clinical neurology.

[4] Hachinski P (1992). Preventable senility: a call for action against the vascular dementias. Lancet.

[5] Ebly EM (1995). Cognitive Impairment in the Nondemented Elderly: Results From the Canadian Study of Health and Aging. Arch Neurol.

[6] Rockwood K, Wentzel C, Hachinski V, Hogan DB, MacKnight C, McDowell I (2000). Prevalence and outcomes of vascular cognitive impairment. Neurology.

[7] Petersen RC, Doody R, Kurz A, Mohs RC, Morris JC, Rabins PV (2001). Current Concepts in Mild Cognitive Impairment. Arch Neurol.

[8] Meyer JS, Xu G, Thornby J, Chowdhury MH, Quach M (2002). Is Mild Cognitive Impairment Prodromal for Vascular Dementia Like Alzheimer's Disease?. Stroke.

[9] Gorelick PB, Scuteri A, Black SE, DeCarli C, Greenberg SM, Iadecola C (2011). Vascular Contributions to Cognitive Impairment and Dementia: A Statement for Healthcare Professionals From the American Heart Association/American Stroke Association. Stroke.

[10] Engelhardt E, Tocquer C, André C, Moreira DM, Okamoto IH, Cavalcanti JLS (2011). Vascular dementia: Cognitive, functional and behavioral assessment. Recommendations of the Scientific Department of Cognitive Neurology and Aging of the Brazilian Academy of Neurology. Part II. Dement Neuropsychol.

[11] Engelhardt E, Tocquer C, André C, Moreira DM, Okamoto IH, Cavalcanti JLS (2011). Vascular dementia: Diagnostic criteria and supplementary exams. Recommendations of the Scientific Department of Cognitive Neurology and Aging of the Brazilian Academy of Neurology. Part I. Dement Neuropsychol.

[12] Brucki SMD, Ferraz AC, Freitas GR, Massaro AR, Radanovic M, Schultz RR (2011). Treatment of vascular dementia Recommendations of the Scientific Department of Cognitive Neurology and Aging of the Brazilian Academy of Neurology. Dement Neuropsychol.

[13] Skrobot OA, O'Brien J.Black S.Chen C.DeCarli C.Erkinjuntti T (2017). The Vascular Impairment of Cognition Classification Consensus Study. Alzheimers Dement.

[14] Sachdev P, Kalaria R, O'Brien J, Skoog I, Alladi S, Black SE (2014). Diagnostic Criteria for Vascular Cognitive Disorders: A VASCOG Statement. Alzheimer Dis Assoc Disord.

[15] American Psychiatric Association (2013). Diagnostic and statistical manual of mental disorders.

[16] Ter Telgte A, Leijsen EMC, Wiegertjes K, Klijn CJM, Tuladhar AM, Leeuw FE (2018). Cerebral small vessel disease: from a focal to a global perspective. Nat Rev Neurol.

[17] Calil V, Elliott E, Borelli WV, Barbosa BJAP, Bram J, Silva FO (2020). Challenges in the diagnosis of dementia: insights from the United Kingdom-Brazil Dementia Workshop. Dement Neuropsychol.

[18] Rizzi L, Rosset I, Roriz-Cruz M (2014). Global epidemiology of dementia: Alzheimer's and vascular types. Biomed Res Int.

[19] Livingston G, Huntley J, Sommerlad A, Ames D, Ballard C, Banerjee S (2020). Dementia prevention, intervention, and care: 2020 report of the Lancet Commission. Lancet.

[20] Jørgensen IF, Aguayo-Orozco A, Lademann M, Brunak S (2020). Age-stratified longitudinal study of Alzheimer's and vascular dementia patients. Alzheimers Dement.

[21] Stephens S, Kenny RA, Rowan E, Allan L, Kalaria RN, Bradbury M (2004). Neuropsychological characteristics of mild vascular cognitive impairment and dementia after stroke. Int J Geriat Psychiatry.

[22] Rasquin SMC, Oostenbrugge RJ, Verhey FRJ, Lodder J (2007). Vascular mild cognitive impairment is highly prevalent after lacunar stroke but does not increase over time: a 2-year follow-up Study. Dement Geriatr Cogn Disord.

[23] Hachinski V, Iadecola C, Petersen RC, Breteler MM, Nyenhuis DL, Black SE (2006). National Institute of Neurological Disorders and Stroke-Canadian Stroke Network Vascular Cognitive Impairment Harmonization Standards. Stroke.

[24] Brucki SMD, Machado MF, Rocha MSG (2012). Vascular Cognitive Impairment (VCI) after non-embolic ischemic stroke during a 12-month follow-up in Brazil. Dement Neuropsychol.

[25] Meguro K, Dodge HH, Anstey K, Peters R (2019). Vascular Mild Cognitive Impairment: Identifying Disease in Community-Dwelling Older Adults, Reducing Risk Factors, and Providing Support. The Osaki-Tajiri and Kurihara Projects. J Alzheimers Dis.

[26] Hughes TF, Liu A, Jacobsen E, Rosano C, Berman SB, Chang CCH (2021). Exercise and the Risk of Mild Cognitive Impairment: Does the Effect Depend on Vascular Factors?. Alzheimer Dis Assoc Disord.

[27] Alonso A, Knopman DS, Gottesman RF, Soliman EZ, Shah AJ, O'Neal WT (2017). Correlates of Dementia and Mild Cognitive Impairment in Patients With Atrial Fibrillation: The Atherosclerosis Risk in Communities Neurocognitive Study (ARIC-NCS). J Am Heart Assoc.

[28] World Health Organization (2019). Risk reduction of cognitive decline and dementia: WHO guidelines.

[29] Suemoto CK, Ferretti-Rebustini REL, Rodriguez RD, Leite REP, Soterio L, Brucki SMD (2017). Neuropathological diagnoses and clinical correlates in older adults in Brazil: A cross-sectional study. PLoS Med.

[30] Gorelick PB, Counts SE, Nyenhuis D (2016). Vascular cognitive impairment and dementia. Biochim Biophys Acta.

[31] Livingston G, Sommerlad A, Orgeta V, Costafreda SG, Huntley J, Ames D (2017). Dementia prevention, intervention, and care. Lancet.

[32] Iadecola C, Duering M, Hachinski V, Joutel A, Pendlebury ST, Schneider JA (2019). Vascular Cognitive Impairment and Dementia. J Am Coll Cardiol.

[33] Grinberg LT (2012). Vascular dementia: current concepts and nomenclature harmonization. Dement Neuropsychol.

[34] Skrobot OA, Attems J, Esiri M, Hortobágyi T, Ironside JW, Kalaria RN (2016). Vascular cognitive impairment neuropathology guidelines (VCING): the contribution of cerebrovascular pathology to cognitive impairment. Brain.

[35] Jellinger KA (2016). Current Pathogenetic Concepts of Vascular Cognitive Impairment. J Neurol Neurol Sci Disord.

[36] Jokinen H, Koikkalainen J, Laakso HM, Melkas S, Nieminen T, Brander A (2020). Global Burden of Small Vessel Disease-Related Brain Changes on MRI Predicts Cognitive and Functional Decline. Stroke.

[37] De Luca C, Virtuoso A, Maggio N, Papa M (2017). Neuro-Coagulopathy Blood Coagulation Factors in Central Nervous System Diseases. Int J Mol Sci.

[38] Rajani RM, Williams A (2017). Endothelial cell-oligodendrocyte interactions in small vessel disease and aging. Clin Sci.

[39] Ungvari Z, Tarantini S, Kiss T, Wren JD, Giles CB, Griffin CT (2018). Endothelial dysfunction and angiogenesis impairment in the ageing vasculature. Nat Rev Cardiol.

[40] Engelhardt E (2014). Brown-Séquard: On neural networks and brain localization of functions. Dement Neuropsychol.

[41] Jellinger KA (2013). Pathology and pathogenesis of vascular cognitive impairment - a critical update. Front Aging Neurosci.

[42] Shin M, Sohn MK, Lee J, Kim DY, Lee SG, Shin YI (2020). Effect of Cognitive Reserve on Risk of Cognitive Impairment and Recovery After Stroke: The KOSCO Study. Stroke.

[43] Mesulam MM (2000). Principles of behavioral and cognitive neurology.

[44] Zekry D, Duyckaerts C, Belmin J, Geoffre C, Herrmann F, Moulias R (2003). The vascular lesions in vascular and mixed dementia: the weight of functional neuroanatomy. Neurobiology of Aging.

[45] Staekenborg SS, Straaten ECW, Flier WM, Lane R, Barkhof F, Scheltens P (2008). Small vessel versus large vessel vascular dementia: Risk factors and MRI findings. J Neurol.

[46] Straaten ECW, Scheltens Ph, Barkhof F (2004). MRI and CT in the diagnosis of vascular dementia. Journal of the Neurological Sciences.

[47] Sudo FK, Amado P, Alves GS, Laks J, Engelhardt E (2017). A continuum of executive function deficits in early subcortical vascular cognitive impairment: A systematic review and meta-analysis. Dement Neuropsychol.

[48] Tullberg M, Fletcher E, DeCarli C, Mungas D, Reed BR, Harvey DJ (2004). White matter lesions impair frontal lobe function regardless of their location. Neurology.

[49] Zhao QL, Zhou Y, Wang YL, Dong KH, Wang YJ (2010). A new diagnostic algorithm for vascular cognitive impairment: the proposed criteria and evaluation of its reliability and validity. Chin Med J (Engl).

[50] Gouw AA, Seewann A, Flier WM, Barkhof F, Rozemuller AM, Scheltens P (2011). Heterogeneity of small vessel disease: a systematic review of MRI and histopathology correlations. J Neurol Neurosurg Psychiatry.

[51] Min ZG, Shan HR, Xu L, Yuan DH, Sheng XX, Xie WC (2021). Diffusion tensor imaging revealed different pathological processes of white matter hyperintensities. BMC Neurol.

[52] McAleese KE, Alafuzoff I, Charidimou A, De Reuck J, Grinberg LT, Hainsworth AH (2016). Post-mortem assessment in vascular dementia: advances and aspirations. BMC Med.

[53] Grinberg LT, Heinsen H (2010). Toward a pathological definition of vascular dementia. J Neurol Sci.

[54] Kalaria RN (2016). Neuropathological diagnosis of vascular cognitive impairment and vascular dementia with implications for Alzheimer's disease. Acta Neuropathol.

[55] Rockwood K, Davis H, MacKnight C, Vandorpe R, Gauthier S, Guzman A (2003). The Consortium to Investigate Vascular Impairment of Cognition: Methods and First Findings. Can J Neurol Sci.

[56] Wardlaw JM, Smith EE, Biessels GJ, Cordonnier C, Fazekas F, Frayne R (2013). Neuroimaging standards for research into small vessel disease and its contribution to ageing and neurodegeneration. Lancet Neurol.

[57] Wardlaw JM, Valdés Hernández MC, Muñoz-Maniega S (2015). What are white matter hyperintensities made of? Relevance to vascular cognitive impairment. J Am Heart Assoc.

[58] Debette S, Schilling S, Duperron MG, Larsson SC, Markus HS (2019). Clinical Significance of Magnetic Resonance Imaging Markers of Vascular Brain Injury: A Systematic Review and Meta-analysis. JAMA Neurol.

[59] Hu HY, Ou YN, Shen XN, Qu Y, Ma YH, Wang ZT (2021). White matter hyperintensities and risks of cognitive impairment and dementia: A systematic review and meta-analysis of 36 prospective studies. Neurosci Biobehav Rev.

[60] Jack CR, Bennett DA, Blennow K, Carrillo MC, Dunn B, Haeberlein SB (2018). NIA-AA Research Framework: Toward a biological definition of Alzheimer's disease. Alzheimers Dement.

[61] Hachinski V, Einhäupl K, Ganten D, Alladi S, Brayne C, Stephan BCM (2019). Preventing dementia by preventing stroke: The Berlin Manifesto. Alzheimers Dement.

[62] Azarpazhooh MR, Hachinski V, Dekosky ST, Asthana S (2019). Handbook of Clinical Neurology: Geriatric Neurology.

[63] World Health Organization (2016). ICD-11 for Mortality and Morbidity Statistics.

[64] Stephan BC, Matthews FE, Khaw KT, Dufouil C, Brayne C (2009). Beyond mild cognitive impairment: vascular cognitive impairment, no dementia (VCIND). Alzheimers Res Ther.

[65] Zanetti M, Ballabio C, Abbate C, Cutaia C, Vergani C, Bergamaschini L (2006). Mild Cognitive Impairment Subtypes and Vascular Dementia in Community-Dwelling Elderly People: A 3-Year Follow-Up Study. J Am Geriatr Soc.

[66] Wentzel C, Rockwood K, MacKnight C, Hachinski V, Hogan DB, Feldman H (2001). Progression of impairment in patients with vascular cognitive impairment without dementia. Neurology.

[67] Madureira S, Verdelho A, Moleiro C, Santos C, Scheltens P, Gouw A (2016). White Matter Changes and Cognitive Decline in a Ten-Year Follow-Up Period: A Pilot Study on a Single-Center Cohort from the Leukoaraiosis and Disability Study. Dement Geriatr Cogn Disord.

[68] Madureira S (2017). Neuropsychological contribution to the study of White Matter Changes: a 10-year longitudinal study.

[69] Palmer K, Wang HX, Bäckman L, Winblad B, Fratiglioni L (2002). Differential Evolution of Cognitive Impairment in Nondemented Older Persons: Results From the Kungsholmen Project. Am J Psychiatry.

[70] Kopchak OO, Bachinskaya NY, Pulyk OR (2020). Vascular risk factors and cognitive functions in the patients with cerebrovascular disease. Wiad Lek.

[71] Wiederkehr S, Laurin D, Simard M, Verreault R, Lindsay J (2009). Vascular Risk Factors and Cognitive Functions in Nondemented Elderly Individuals. J Geriatr Psychiatry Neurol.

[72] O'Brien JT, Erkinjuntti T, Reisberg B, Roman G, Sawada T, Pantoni L (2003). Vascular cognitive impairment. Lancet Neurol.

[73] Graff-Radford J (2019). Vascular Cognitive Impairment. Continuum (Minneap Minn).

[74] Arvanitakis Z, Leurgans SE, Wang Z, Wilson RS, Bennett DA, Schneider JA (2011). Cerebral amyloid angiopathy pathology and cognitive domains in older persons. Ann Neurol.

[75] Oh ES, Rabins PV (2019). Dementia. Ann Intern Med.

[76] Ramos AM, Stein AT, Castro ED, Chaves MLF, Okamato I, Nitrini R (2009). Demência do Idoso: diagnóstico na Atenção Primária.

[77] Patnode CD, Perdue LA, Rossom RC, Rushkin MC, Redmond N, Thomas RG (2020). Screening for Cognitive Impairment in Older Adults: An Evidence Update for the U.S. Preventive Services Task Force.

[78] Creavin ST, Wisniewski S, Noel-Storr AH, Trevelyan CM, Hampton T, Rayment D (2016). Mini-Mental State Examination (MMSE) for the detection of dementia in clinically unevaluated people aged 65 and over in community and primary care populations. Cochrane Database Syst Rev.

[79] Nitrini R, Lefèvre BH, Mathias SC, Caramelli P, Carrilho PEM, Sauaia N (1994). Testes neuropsicológicos de aplicação simples para o diagnóstico de demência. Arq Neuropsiquiatr.

[80] Nitrini R, Bucki SMD, Yassuda MS, Fichman HC, Caramelli P (2021). The Figure Memory Test: diagnosis of memory impairment in populations with heterogeneous educational background. Dement Neuropsychol.

[81] Nitrini R, Caramelli P, Porto CS, Charchat-Fichman H, Formigoni AP, Carthery-Goulart MT (2007). Brief cognitive battery in the diagnosis of mild Alzheimer's disease in subjects with medium and high levels of education. Dement Neuropsychol.

[82] Borson S, Scanlan J, Brush M, Vitaliano P, Dokmak A (2000). The mini-cog: a cognitive “vital signs” measure for dementia screening in multi-lingual elderly. Int J Geriatr Psychiatry.

[83] Nasreddine ZS, Phillips NA, Bédirian V, Charbonneau S, Whitehead V, Collin I (2005). The Montreal Cognitive Assessment, MoCA: a brief screening tool for mild cognitive impairment. J Am Geriatr Soc.

[84] Roalf DR, Moberg PJ, Xie SX, Wolk DA, Moelter ST, Arnold SE (2013). Comparative accuracies of two common screening instruments for classification of Alzheimer's disease, mild cognitive impairment, and healthy aging. Alzheimers Dement.

[85] Saczynski JS, Inouye SK, Guess J, Jones RN, Fong TG, Nemeth E (2015). The Montreal Cognitive Assessment: Creating a Crosswalk with the Mini-Mental State Examination. J Am Geriatr Soc.

[86] Seitz DP, Chan CC, Newton HT, Gill SS, Herrmann N, Smailagic N (2018). Mini-Cog for the diagnosis of Alzheimer's disease dementia and other dementias within a primary care setting. Cochrane Database Syst Rev.

[87] Ghafar MZAA, Miptah HN, O'Caoimh R (2019). Cognitive screening instruments to identify vascular cognitive impairment: A systematic review. Int J Geriatr Psychiatry.

[88] Gilly Nardy B (2019). Instrumentos de rastreio para o Comprometimento Cognitivo Vascular Subcortical: Revisão da literatura e adaptação do Brief Memory and Executive Test (BMET) ao contexto brasileiro.

[89] Ylikoski R, Jokinen H, Andersen P, Salonen O, Madureira S, Ferro J (2007). Comparison of the Alzheimer's Disease Assessment Scale Cognitive Subscale and the Vascular Dementia Assessment Scale in differentiating elderly individuals with different degrees of white matter changes. The LADIS Study. Dement Geriatr Cogn Disord.

[90] Hong YJ, Yoon B, Shim YS, Han IW, Han SH, Park KH (2014). Do Alzheimer's disease (AD) and subcortical ischemic vascular dementia (SIVD) progress differently?. Arch Gerontol Geriatr.

[91] Pendlebury ST, Cuthbertson FC, Welch SJV, Mehta Z, Rothwell PM (2010). Underestimation of cognitive impairment by Mini-Mental State Examination versus the Montreal Cognitive Assessment in patients with transient ischemic attack and stroke: a population-based study. Stroke.

[92] Webb AJS, Pendlebury ST, Li L, Simoni M, Lovett N, Mehta Z (2014). Validation of the Montreal cognitive assessment versus mini-mental state examination against hypertension and hypertensive arteriopathy after transient ischemic attack or minor stroke. Stroke.

[93] Pendlebury ST, Mariz J, Bull L, Mehta Z, Rothwell PM (2012). MoCA, ACE-R, and MMSE versus the National Institute of Neurological Disorders and Stroke-Canadian Stroke Network Vascular Cognitive Impairment Harmonization Standards Neuropsychological Battery after TIA and stroke. Stroke.

[94] Mioshi E, Dawson K, Mitchell J, Arnold R, Hodges JR (2006). The Addenbrooke's Cognitive Examination Revised (ACE-R): a brief cognitive test battery for dementia screening. Int J Geriatr Psychiatry.

[95] César KG, Yassuda MS, Porto FHG, Brucki SMD, Nitrini R (2017). Addenbrooke's cognitive examination-revised: normative and accuracy data for seniors with heterogeneous educational level in Brazil. Int Psychogeriatr.

[96] César KG, Yassuda MS, Porto FHG, Brucki SMD, Nitrini R (2019). MoCA Test: normative and diagnostic accuracy data for seniors with heterogeneous educational levels in Brazil. Arq Neuropsiquiatr.

[97] Moreira IFH, Lourenço RA, Soares C, Engelhardt E, Laks J (2009). Cambridge Cognitive Examination: performance of healthy elderly Brazilians with low education levels. Cad Saude Publica.

[98] Moreira IFH, Bezerra AB, Sudo FK, Alves GS, Ericeira-Valente L, Tiel C (2013). CAMCOG - valores das subescalas em idosos normais com níveis diferentes de escolaridade. Aspectos preliminares. Rev Bras Neurol.

[99] Chaves MLF, Camozzato AL, Godinho C, Kochhann R, Schuh A, Almeida VL (2007). Validity of the clinical dementia rating scale for the detection and staging of dementia in Brazilian patients. Alzheimer Dis Assoc Disord.

[100] Sudo FK, Alves GS, Moreira DM, Laks J, Engelhardt E (2016). Subcortical Vascular Cognitive Impairment staged through cdr's functional subsum (cdr-func): Preliminary results from an outpatient sample. eNeurologicalSci.

[101] Flier WM, Skoog I, Schneider JA, Pantoni L, Mok V, Chen CLH (2018). Vascular cognitive impairment. Nat Rev Dis Primers.

[102] Verdelho A, Wardlaw J, Pavlovic A, Pantoni L, Godefroy O, Duering M (2021). Cognitive impairment in patients with cerebrovascular disease: A white paper from the links between stroke ESO Dementia Committee. Eur Stroke J.

[103] Consoli A, Pasi M, Pantoni L (2012). Vascular mild cognitive impairment: concept, definition, and directions for future studies. Aging Clin Exp Res.

[104] Poggesi A, Salvadori E, Pantoni L, Pracucci G, Cesari F, Chiti A (2012). Risk and Determinants of Dementia in Patients with Mild Cognitive Impairment and Brain Subcortical Vascular Changes: A Study of Clinical, Neuroimaging, and Biological Markers-The VMCI-Tuscany Study: Rationale, Design, and Methodology. Int J Alzheimers Dis.

[105] Sudo FK, Alves GS, Tiel C, Ericeira-Valente L, Moreira DM, Laks J (2015). Neuroimaging criteria and cognitive performance in vascular mild cognitive impairment: A systematic review. Dement Neuropsychol.

[106] Harper L, Barkhof F, Fox NC, Schott JM (2015). Using visual rating to diagnose dementia: a critical evaluation of MRI atrophy scales. J Neurol Neurosurg Psychiatry.

[107] Fazekas F, Chawluk JB, Alavi A, Hurtig HI, Zimmerman RA (1987). MR signal abnormalities at 1 5 T in Alzheimer's dementia and normal aging. AJR Am J Roentgenol.

[108] Pantoni L, Basile AM, Pracucci G, Asplund K, Bogousslavsky J, Chabriat H (2005). Impact of age-related cerebral white matter changes on the transition to disability - the LADIS study: rationale, design and methodology. Neuroepidemiology.

[109] Scheltens P, Barkhof F, Leys D, Pruvo JP, Nauta JJ, Vermersch P (1993). A semiquantative rating scale for the assessment of signal hyperintensities on magnetic resonance imaging. J Neurol Sci.

[110] Wahlund LO, Barkhof F, Fazekas F, Bronge L, Augustin M, Sjögren M (2001). A new rating scale for age-related white matter changes applicable to MRI and CT. Stroke.

[111] Schmidt R, Schmidt H, Haybaeck J, Loitfelder M, Weis S, Cavalieri M (2011). Heterogeneity in age-related white matter changes. Acta Neuropathol.

[112] Chowdhury MH, Nagai A, Bokura H, Nakamura E, Kobayashi S, Yamaguchi S (2011). Age-Related Changes in White Matter Lesions, Hippocampal Atrophy, and Cerebral Microbleeds in Healthy Subjects Without Major Cerebrovascular Risk Factors. J Stroke Cerebrovasc Dis.

[113] Kandel BM, Avants BB, Gee JC, McMillan CT, Erus G, Doshi J (2016). White matter hyperintensities are more highly associated with preclinical Alzheimer's disease than imaging and cognitive markers of neurodegeneration. Alzheimers Dement (Amst).

[114] Havakuk O, King KS, Grazette L, Yoon AJ, Fong M, Bregman N (2017). Heart Failure-Induced Brain Injury. J Am Coll Cardiol.

[115] Doehner W, Ural D, Haeusler KG, Celutkien J, Bestetti R, Cavusoglu Y (2018). Heart and brain interaction in patients with heart failure: overview and proposal for a taxonomy. A position paper from the Study Group on Heart and Brain Interaction of the Heart Failure Association: Heart and brain interaction in heart failure. Eur J Heart Fail.

[116] Passiak BS, Liu D, Kresge HA, Cambronero FE, Pechman KR, Osborn KE (2019). Perivascular spaces contribute to cognition beyond other small vessel disease markers. Neurology.

[117] Teixeira MM, Passos VMA, Barreto SM, Schmidt MI, Duncan BB, Beleigoli AMR (2020). Association between diabetes and cognitive function at baseline in the Brazilian Longitudinal Study of Adult Health (ELSA-Brasil). Sci Rep.

